# Tracing the Derivation of Embryonic Stem Cells from the Inner Cell Mass by Single-Cell RNA-Seq Analysis

**DOI:** 10.1016/j.stem.2010.03.015

**Published:** 2010-05-07

**Authors:** Fuchou Tang, Catalin Barbacioru, Siqin Bao, Caroline Lee, Ellen Nordman, Xiaohui Wang, Kaiqin Lao, M. Azim Surani

**Affiliations:** 1 Wellcome Trust/Cancer Research UK Gurdon Institute of Cancer and Developmental Biology, University of Cambridge, Tennis Court Road, Cambridge CB2 1QN, UK; 2 Genetic Systems, Applied Biosystems, part of Life Technologies, 850 Lincoln Centre Drive, Foster City, CA 94404, USA

**Keywords:** STEMCELL, RNA

## Abstract

During the transition from the inner cell mass (ICM) cells of blastocysts to pluripotent embryonic stem cells (ESCs) in vitro, a normal developmental program is replaced in cells that acquire a capacity for infinite self-renewal and pluripotency. We explored the underlying mechanism of this switch by using RNA-Seq transcriptome analysis at the resolution of single cells. We detected significant molecular transitions and major changes in transcript variants, which include genes for general metabolism. Furthermore, the expression of repressive epigenetic regulators increased with a concomitant decrease in gene activators that might be necessary to sustain the inherent plasticity of ESCs. Furthermore, we detected changes in microRNAs (miRNAs), with one set that targets early differentiation genes while another set targets pluripotency genes to maintain the unique ESC epigenotype. Such genetic and epigenetic events may contribute to a switch from a normal developmental program in adult cells during the formation of diseased tissues, including cancers.

## Highlights

► Single-cell RNA-Seq analysis during ICM to ESC reveals molecular switches ► Enhanced repressive epigenetic factors may sustain ESC epigenotype ► Changes in miRNAs targets differentiation and pluripotency mRNAs ► Transcript variants and genes for metabolism enhanced during ESC formation

## Introduction

The derivation of embryonic stem cells (ESCs) from the inner cell mass (ICM) of mouse blastocysts consisting of about 20 cells occurs in vitro under a variety of culture conditions, such as in the presence of leukemia inhibitory factor (LIF) and fetal calf serum (FCS) (Evans and Kaufman, 1981; Ying et al., 2008). After about 5 days in culture, the inner cell mass outgrowths of blastocysts are disrupted into small clusters of cells and passaged until the establishment of ESC lines. Thus, the ICM cells that, in vivo, are subject to a strict developmental program undergo a transformation into cells with a capacity for infinite self-renewal while retaining pluripotency. The precise molecular changes accompanying this transition remain to be fully elucidated, which is hampered by the limited number of cells available for analysis (Niwa, 2007).

Pluripotent E3.5-E4.5 primitive ectoderm/epiblast (PE) and ESCs can both contribute to all three germ layers and the germ line when injected into host blastocysts to form chimera (Niwa, 2007). However, only the ESCs cultured in vitro have the capacity for unlimited self-renewal while retaining their pluripotency (Niwa, 2007; Smith, 2006). Some differences between the ICM and ESCs have been identified, such as the expression of pramel5, pramel6, and pramel7 in the ICM, which are repressed in ESC (Kaji et al., 2007). Other genes, including Dicer (Bernstein et al., 2003; Kanellopoulou et al., 2005; Murchison et al., 2005), Nanog (Chambers et al., 2007; Chambers et al., 2003; Mitsui et al., 2003), Mbd3 (Kaji et al., 2006; Kaji et al., 2007), and Ezh2 (O'Carroll et al., 2001; Shen et al., 2008), are essential for the establishment of pluripotent PE cells in the ICM but dispensable for the maintenance of ESCs.

There have been intensive studies on ESCs in recent years, but these have usually been on bulk cells by RNA-Seq, cDNA microarray, SAGE, and EST sequencing (Niwa, 2007; Ivanova et al., 2006; Cloonan et al., 2008). However, the precise changes accompanying the process of conversion of ICM to ESCs remain to be fully elucidated. To gain insight into this process, we used blastocysts from Oct4-ΔPE-GFP transgenic mice and cultured them in vitro under the classical conditions consisting of LIF and FCS used for the derivation of ESCs (Niwa, 2007). The Oct4-ΔPE-GFP reporter we used is under the control of only the distal enhancer for Oct4 (also known as Pou5f1) and lacks the proximal enhancer (Yeom et al., 1996). This GFP reporter shows expression in the E3.5 ICM, E4.5 epiblast, primordial germ cells (PGCs), and ESCs, but not in the postimplantation epiblast or in the epiblast stem cells (EpiSCs) (Yeom et al., 1996; Bao et al., 2009). Notably, the distal enhancer of Oct4 represents the densest binding locus for the key pluripotency-specific transcription factors in ESCs (Chen et al., 2008), which makes it an ideal reporter for tracing the course of changes during the establishment of ESCs from ICM. By analyzing single Oct4-ΔPE-GFP-positive and Oct4-ΔPE-GFP-negative cells, we set out to monitor changes in ICM cells during their progression toward ESCs. We used our recently developed single-cell RNA-Seq transcriptome analysis to investigate the critical early changes during this process (Tang et al., 2009).

## Results and Discussion

### Analysis of Individual ICM Outgrowth Cells

**Figure 1:**
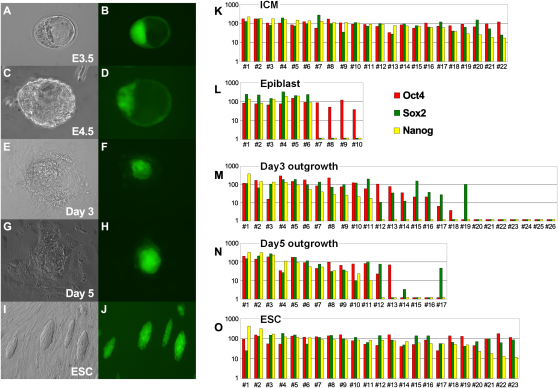
Morphology of ICM Outgrowth Bright field and fluorescence image of mouse E3.5 blastocyst (day 0 ICM outgrowth) (A and B), E4.5 blastocyst (C and D), day 3 ICM outgrowth (E and F), day 5 ICM outgrowth (G and H), and ESCs (I and J); real-time PCR measured gene expression in single cells of ICM outgrowth: *Oct4*, *Sox2*, and *Nanog* expression in 22 single E3.5 ICM cells (K), in 10 single E4.5 epiblast cells (L), in 26 single day 3 ICM outgrowth cells (M), in 17 single day 5 ICM outgrowth cells (N), and in 23 single ESCs (O). The y axis is normalized expression levels based on the mean expression values of all single cells for the given gene.

First, we analyzed the three key pluripotency genes during the course of blastocyst culture and the formation of outgrowths (Figure 1). At each stage, we chose between 10 and 26 single cells for analysis. We generated cDNAs by whole transcriptome amplification (WTA) of these individual cells (see Experimental Procedures for details). All ICM cells (22/22) tested showed high expression of *Oct4*, *Sox2*, and *Nanog*. However, among cells from day 3 outgrowths that had high *Oct4* expression, about 39% (7/18) had already lost expression of *Nanog* and/or *Sox2*, indicating that they might be losing pluripotency. By contrast, most of the cells from day 5 outgrowths (11/13) that had high *Oct4* expression also showed high expression of both *Sox2* and *Nanog*, suggesting that these may represent the earliest population that had acquired or were likely on course to acquire the ESC-like fate with the potential for self-renewal. We were also able to establish an ESC line from a single cell isolated from a day 5 outgrowth (data not shown). As expected, all the ESCs (23/23) had high expression of these three pluripotency genes.

### Expression Dynamics of 385 Genes in 74 Single Cells from ICM to ESCs

**Figure 2:**
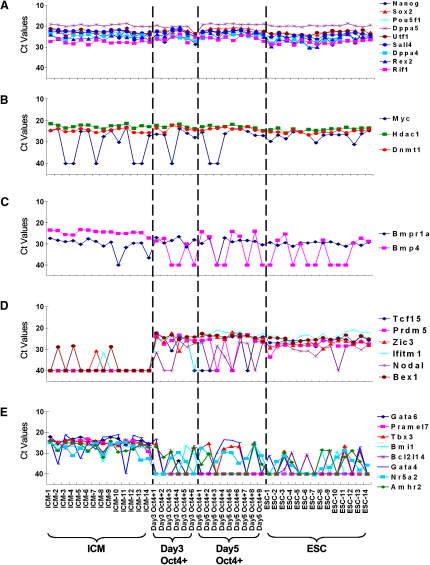
Gene Expression Measured by Real-Time PCR Gene expression measured by real-time PCR in single cells of fourteen ICM (E3.5), six day 3 ICM outgrowth cells, nine day 5 ICM outgrowth cells, and 14 ESCs (Table S1).

Next, we chose 385 pluripotency and early differentiation related genes to monitor their expression in cells from the ICM, as well as from day 3 and day 5 outgrowths, and from ESCs at single-cell resolution (Table S1). All 14 ESCs analyzed had high expression (Ct = 19–28) of *Oct4*, *Sox2*, *Nanog*, *Dppa4*, *Dppa5*, *Sall4*, *Utf1*, *Rex2*, and *Rif1*, indicating their pluripotent character (Figure 2A and Figure S1). By contrast, we detected little or no expression (Ct = 40) of all 23 early differentiation marker genes (ectoderm markers: *Pax6*, *Otx1*, *Neurod1*, *Nes*, *Lhx5*, and *Hoxb1*; mesoderm markers: *Tbx2*, *T*, *Nkx2-5*, *Myod1*, *Myf5*, *Mesdc1*, *Mesdc2*, *Kdr*, *Isl1*, *Hand1*, and *Eomes*; endoderm markers: *Onecut1*, *Gata4*, *Gata5*, and *Gata6*; extraembryonic markers: *Cdx2* and *Tpbpa*) (see Table S1). Similarly, all 14 cells isolated and analyzed from ICM showed high expression of the nine pluripotency-specific genes. However, expression of some genes, for example, *c-Myc*, which was shown to be an important reprogramming factor for pluripotency (Takahashi and Yamanaka, 2006), was highly heterogeneous in cells from the ICM (Ct = 24–40); this variability was progressively reduced until, finally, all ESCs consistently expressed c-*Myc* (Figure 2B). Interestingly, we found that *Tet1* and *Tet2* (Table S1), which were recently shown to mediate DNA demethylation in ESCs, were highly expressed in both ICM and ESCs, but their expression only decreased in Oct4-negative cells present in the ICM outgrowths. Thus, our observations support their importance for pluripotency (Tahiliani et al., 2009).

Since ESCs can also be maintained in an undifferentiated state by LIF and BMP4 (Ying et al., 2003), we investigated the expression of a key receptor, *Bmpr1a*, and found it to be heterogeneous in the ICM (Ct = 27–40). However during the ICM outgrowth, *Bmpr1a* expression was detected more consistently until, finally, all ESCs (14/14) showed strong expression. This suggests that all ESCs have the potential to respond to Bmp4 signaling (Figure 2C). Conversely, for *Bmp4*, all ICM cells (14/14) showed high expression, but this declined during the course of ICM outgrowths so that ultimately only about 50% (7/14) of individual ESCs retained *Bmp4* expression (Ct = 25–40). This is compatible with the fact that maintenance of ESCs can be achieved by the addition of exogenous Bmp4 or serum, which contains Bmp4 (Ying et al., 2003).

During the course of ICM outgrowth toward ESCs, we found clear upregulation of several genes, including *Tcf15*, *Prdm5*, *Zic3*, *Ifitm1*, *Nodal*, and *Bex1*, indicating that they may potentially be important during the transition to ESCs and/or for their subsequent maintenance (Figure 2D). Indeed, *Nodal* is a known regulator of self-renewal but is not essential for the pluripotency of ESCs (see below). By contrast, there was clear downregulation of some genes during ICM outgrowth, such as *Gata4*, *Gata6*, *Pramel7*, *Tbx3*, *Bmi1*, *Bcl2l14*, *Nr5a2*, and *Amhr2*, which potentially have ICM specific development-related functions (Figure 2E). For example, ICM has the potential to develop into primitive endoderm cells, for which *Gata4* and *Gata6* are crucial regulators (Fujikura et al., 2002; Koutsourakis et al., 1999; Morrisey et al., 1998). Thus, repression of these genes may allow ICM cells to exit from their inherent developmental program as they acquire the ability for self-renewal while retaining pluripotency as ESCs.

### Molecular Changes during the Transition from ICM to ESCs

**Figure 3:**
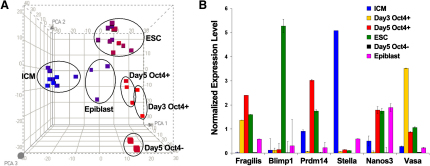
Transcriptome Analysis of ICM Outgrowth Cells (A) The principal component analysis of ICM outgrowth cells. The nine E3.5 ICM, three E4.5 Epiblast, two day 3 Oct4^+^Sox2^+^Nanog^+^ outgrowth cells, three day 5 Oct4^+^Sox2^+^Nanog^+^ outgrowth cells, two day 5 Oct4^−^Sox2^−^Nanog^−^ outgrowth cells, and twelve ESCs are independently clustered (Table S2). (B) Expression dynamics of marker genes of early primordial germ cells (PGCs). Averaged expression of different individual cells was shown. The error bar represents the coefficient of variation (CV) between individual cells.

To understand the dynamic nature of gene expression in individual cells at the whole-genome scale, we randomly selected 12 individual ESCs and generated their digital transcriptome profile (Figure 3A, Figure S2, and Tables S2 and S3) (Tang et al., 2009). Indeed, all of the 12 ESCs analyzed had high expression of *Oct4*, *Sox2*, *Nanog*, *Rex1* (also known as *Zfp42*), *Dppa5*, and *Utf1*, which indicates that all of them are in an undifferentiated state and are pluripotent. To confirm the reliability of our single-cell RNA-Seq approach, we compared our data with that obtained from bulk analysis of ESCs (Cloonan et al., 2008). We found that on average, an individual ESC expresses 10,815 genes (RPM > 0.1), which means that we captured expression of at least 94.6% of the genes in a single cell of those detected by deep sequencing in bulk assays of ESCs (Cloonan et al., 2008). Overall, 65.8% (13,326 out of 20,259) of known genes were expressed in 12 single ESCs, which shows that our RNA-Seq data represent an accurate reflection of the entire transcriptome in ESCs at single-cell resolution.

To understand the relationship between ESCs and the ICM/Epiblast cells from which they were derived, we compared the single-cell RNA-Seq transcriptomes of these cells (Figure S2) to determine the extent to which ESCs resemble E3.5 ICM or E4.5 Epiblast cells (Nichols et al., 2009). We found that the molecular signature of all undifferentiated ESCs maintained under our culture conditions are clearly different from both ICM and epiblast cells based on the principal component analysis of their transcriptomes. This means that at the molecular level, ESCs are distinct from E3.5 ICM or E4.5 Epiblast (Figure 3A). We detected a large set of genes, which show clear differential expression between ICM/Epiblast and ESCs. (Table S2 and Figure S3. Note 2,475 genes with fold change, FC[ESC/ICM] > 4, p < 0.01, and 2,362 genes with FC[ESC/ICM] < 0.25, p < 0.01; 2,110 genes with FC[ESC/Epiblast] > 4, p < 0.01 and 1,170 genes with FC[ESC/Epiblast] < 0.25, p < 0.01).

**Figure 4:**
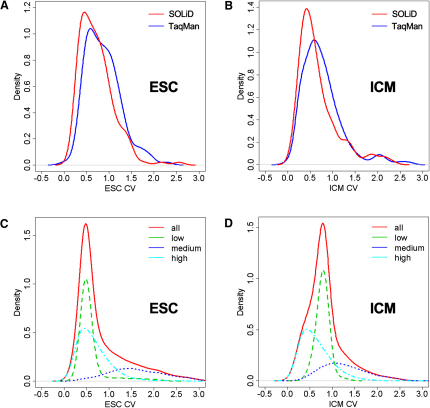
Plots of the Distribution of Coefficient of Variation between Individual Cells 158 (A) and 117 (B) genes are detected by TaqMan real-time PCR (Ct < 32 in at least half of the cells) in ESC and ICM cells, respectively, and used to compare cell-to-cell measurement variance. Density of coefficients of variation (CVs) for Ct measurements (blue) and RPM measurements representing RNA-Seq transcripts counts per million reads (red) across both ESC (A) and ICM (B) show a small difference between the two platforms. A similar representation is generated using the entire set of transcripts (24,435) separated into three categories: highly expressed genes (RPM > 10, aqua blue), mid-expressed genes (1 < PRM < 10, blue), and low-expressed genes (RPM < 1, green). CV density of each group of transcripts is represented for ESC (C) and ICM (D). The red curves are the sum of high-, mid- and low-expressed genes density of CVs. The mid-expressed genes tend to have higher cell-to-cell variations (Table S2). For each transcript, RPMs were used to calculate mean and standard deviation across cells of the same type. CV was defined as the ratio between the standard deviation and the mean value.

We found that genes expressed at the mid levels (read per million reads, 1 < RPM < 10), showed higher cell-to-cell variations than the ones expressed at high levels (RPM > 10, Figure 4), which indicates that the former set of genes have a higher propensity for a more dynamic regulation of expression among individual cells of the same type. These genes include *Hoxd13*, *Hoxb3*, *Hoxb5*, and *Ddx3y* that showed highly variable expression in ESCs, whereas *Gm364*, *Tmem80*, *Hdx*, *Trpm3*, *Enox2*, *Ilvbl*, *Has3*, *Pygm*, and *Fbxw13* showed a great variation in expression within ICM cells. Some genes, such as *Tnk1*, *Myof*, *Adamts9*, *Tspan12*, *Rhox6*, *Epha7*, *Dhrs3*, *Fam189a1*, and *Nudt18*, showed highly variable expression in both ESCs and ICM (Table S2). These variations are probably not because of technical reasons because genes expressed at low levels (RPM < 1) showed less cell-to-cell variation compared to genes expressed at mid levels. For these genes with large coefficient of variations (CV > 1) between cells of ESCs and ICM, Gene Ontology (GO) analysis showed that the genes involved in cellular growth, cellular assembly, amino acid metabolism, and lipid metabolism were significantly enriched (p < 0.0001).

Next, we wanted to establish what general trends exist in gene expression changes during the transition from ICM to ESCs. GO analysis showed that for genes that changed dramatically in their expression from ICM to ESCs (FC[ESC/ICM] > 4, or < 0.25, p < 0.01), we found strong enrichment of genes for transcription-related processes, such as DNA-dependent regulation of transcription (enrichment > 1.5, p < 2.5 × 10^−14^), transcription factor activity (enrichment > 1.45, p < 2.6 × 10^−11^), transcription regulator activity (enrichment > 1.51, p < 4.2 × 10^−9^), positive regulation of transcription from RNA polymerase II promoter (enrichment > 1.51, p < 2.0 × 10^−7^), and transcription repressor activity (enrichment > 1.4, p < 1.8 × 10^−5^, Table S4). For example, 29 out of the 34 genes of the nucleic acid metabolism pathway and 25 out of the 32 genes of lipid metabolism pathway showed dramatic changes in their expression between ICM and ESCs (Figures S5A and S5B). We also found clear enrichment of cell-fate-related pathways, such as cellular development (24 out 31 genes), organismal development (27 out of 33 genes), and cellular assembly and organization (32 out of 35 genes), indicating a shift in the developmental potential from ICM to ESCs. On the other hand, we found significant similarities in signaling-related processes (including signal transduction, signal transducer activity, receptor activity, G protein coupled receptor activity, and G protein coupled receptor protein signaling pathway), indicating that their expression is relatively comparable in ICM and ESCs (Table S4). However, ESCs have the ability to self-renew, whereas ICM does not. Thus, the 4,837 genes differentially expressed between ICM and ESCs might provide insights into genes that regulate self-renewal ability and unique metabolism in ESCs. To confirm the reliability of our observations on differentially expressed genes by our single-cell RNA-Seq, we compared 108 genes that were detected by both real-time PCR (Ct < 32) and RNA-Seq (>0.1 RPM) and found that their correlation coefficient is 0.92, confirming the accuracy of our single-cell RNA-Seq data (Figure S3).

### Alternative Splicing during the ICM Outgrowth at Whole-Genome Scale

**Figure 5:**
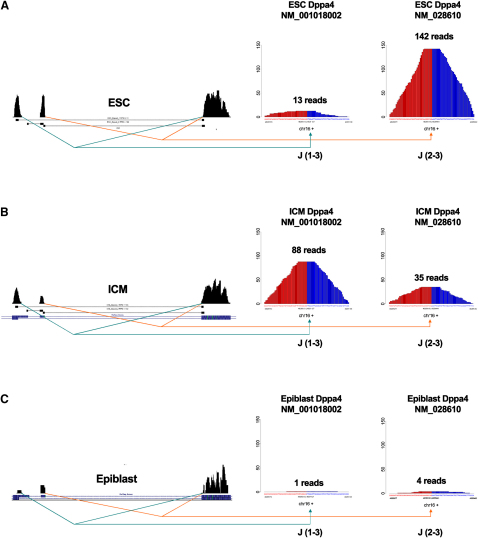
Splice-Specific Differential Expression The coverage plots of two junctions in (A) ESC, (B) E3.5 ICM, and (C) E4.5 epiblast of Dppa4 gene. The junction counts of *Dppa4* transcript variant no. 1 (NM_001018002) are 12.47-fold more in ICM (88 reads, RPM = 150.16) than that in ESC (13 reads, RPM = 12.03), while the junction counts of *Dppa4* transcript variant no. 2 (NM_028610) are 2.2-fold less in the ICM (35 reads, RPM = 59.72) than that in ESC (142 reads, RPM = 131.43, Table S2). RPM, read per million aligned reads.

Alternative splicing plays an important role in defining tissue identity and specificity. It is estimated that nearly 95% of the mammalian multiexon genes express multiple transcript variants through alternative splicing (Chen and Manley, 2009). We wished to know if alternative splicing was a major feature during the outgrowth process of ICM toward ESCs. We addressed the expression dynamics of all the 6,331 transcript variants from the 2,567 RefSeq genes with multiple known isoforms, which has not been addressed previously. 1,852 transcript variants were expressed (at least 5 counts) in either ICM or ESCs. And from them, 417 transcript variants were upregulated (fold change, FC[ESC/ICM, splicing] > 2, p < 0.01), and 586 transcript variants were clearly downregulated (FC[ESC/ICM, splicing] < 0.5, p < 0.01) (Figure 5; Table S2). Thus, there was a dramatic change in 54.2% (1,003 out of 1,852) of the expressed transcript isoforms during ICM outgrowth. Interestingly, we found that epigenetic regulators, such as transcript variants of heterochromatin binding protein *Cbx5* (also known as *Hp1a*), *Setdb1* (also known as *Eset*), *Suv39h2*, *Ehmt2* (also known as *G9a*), *Suv12*, *Kdm4a* (also known as *Jmjd3*), *Sirt2*, *Smarcb1*, and *Kat2a* (also known as *Gcn5*), showed between 2- and 132-fold change during ICM outgrowth. More importantly, there were 128 transcript variants expressed in ICM cells that were completely lost in ESCs. Conversely, 169 variants that were not expressed in ICM were clearly expressed in ESCs (Figure S4). Correspondingly, the expression of tissue-specific alternative splicing factors strongly changed (5- to 60-fold) during ICM outgrowth, such as *nPTB* (also known as *Ptbp2*), *Rbm35b* (also known as *Esrp2*), *Tia1*, *Slm2* (also known as *Khdrbs3*), *Celf4* (also known as *Brunol4*), and *Celf5* (also known as *Brunol5*). Thus, there were global changes in the regulation of alternative splicing during ICM outgrowth.

GO analysis of transcription variants showed that the general cell metabolism-related processes, such as mRNA processing (enrichment > 1.41, p < 0.02), RNA splicing (enrichment > 1.56, p < 0.015), protein modification processes (enrichment > 2.41, p < 0.0037), cytoskeleton (enrichment > 1.27, p < 0.007), microtubule (enrichment > 1.57, p < 0.008), carbohydrate metabolic processes (enrichment > 1.65, p < 0.03), modification-dependent protein catabolic processes (enrichment > 1.3, p < 0.03), are clearly enriched for these transcript variants (Table S4). This suggests that one of the main sources of global regulation of alternative splicing from ICM to ESCs probably reflects the accommodation of ICM cells from in vivo embryonic environment to ESC in vitro culture condition, which might be important for the regulation of basal cell metabolism. Furthermore, cell-cycle-related processes (including cell division and mitosis) are also enriched in these significantly up- or downregulated transcript variants, indicating the potential differences in cell-cycle regulation between ICM and ESCs.

### Expression Dynamics of Epigenetic Regulators during the ICM Outgrowth

Since the process of ICM outgrowths and formation of ESCs involves arrest of a normal developmental program and initiation of self-renewal while retaining pluripotency, it is likely that epigenetic regulators may have an important role during this process. For this reason, we analyzed the expression of 114 known key epigenetic regulators (Table S4). We found that 37 of them showed strong upregulation and 16 of them were clearly downregulated. Thus 46.5% (53 out of 114) of known epigenetic regulators showed changes in expression, which may be necessary to underpin the phenotypic changes in these cells. More importantly, the majority of the epigenetic regulators that show increased expression are linked to a repressive epigenetic status. Thus, for DNA methylation, *Dnmt3a*, *Dnmt3b*, *Dnmt3l*, *Mecp2*, and *Mbd2* increased between 2- and 12-fold from ICM to ESCs. Similarly, histone deacetylases *Hdac5*, *Hdac6*, *Hdac7*, *Hdac11*, H3K9 methyltransferase *Ehmt1* (also known as *Glp*), H4K20 methyltransferase *Suv420h2*, and heterochromatin binding protein *Cbx1* (also known as *Hp1b*) increased from 2- to 40-fold. Conversely, a large proportion of the epigenetic modifiers known to confer an active epigenetic status were downregulated. These include histone acetyltransferases *Ncoa3* (also known as *ACTR*), *Crebbp* (also known as *CBP/P300*), *Clock*, H3K9 demethylases *Kdm4a* (also known as *Jhdm3a*), *Kdm4d* (also known as *Jmjd2d*), H3K27 demethylase *Kdm6b* (also known as *Jmjd3*), and H3K4 methyltransferase *Mll3* (also known as *Kmt2c*), which showed between 2- and 10-fold downregulation from ICM outgrowth to ESCs.

These results indicate that ESCs probably have a globally more repressive epigenetic status compared with the ICM. Since ICM cells in vivo undergo rapid and transient phenotypic and developmental changes in the course of early postimplantation development, these cells may require a greater epigenetic flexibility. By contrast, the more repressive epigenetic status of ESCs is probably important for the maintenance and propagation of their undifferentiated pluripotent state while retaining the capacity for infinite self-renewal.

### Correlation between Gene Expression, Pluripotency, Cell Differentiation, and Cell Fate

Next, we examined in greater detail changes in gene expression to further assess the differences between ICM and ESCs (Figure S2). Whereas ESCs and ICM are both pluripotent, the phenotype of ESCs is relatively constant as they undergo self-renewal, while ICM cells cannot do so in vivo because they are poised to undergo further changes according to their developmental program (Smith, 2006; Niwa, 2007). From this analysis, we found four clusters of interesting genes related to pluripotency and self-renewal (Figure S6).

The first cluster of genes is upregulated during ICM outgrowth, such as *Nodal*, *Eras*, *Lin28*, *Smad1*, *Zic3*, *Id1*, *Id2*, *Tcf3*, *Kit* (also known as *c-Kit*), *Kitl* (also known as *Scf*), *Prdm5*, *Prdm16*, *Klf12*, *Zfp41*, *Sox3*, *Ifitm3* (also known as *Fragilis*), *Pim2*, *Cdh3* (also known as *P-cadherin*), and *Nr0b1* (also known as *Dax1*); these genes correlate positively with the capacity for self-renewal exhibited by ESCs. Indeed, this trend is confirmed by the analysis of cells in day 5 ICM outgrowths that have ceased to express pluripotency genes, where this first cluster of genes is downregulated (Figure S6A). While some of the genes in this cluster were shown to be important for pluripotency, we suggest that at least some of them are probably also important for self-renewal. In fact, *Eras* and *Nodal* that were recovered in this cluster are two known regulators that are crucial for the self-renewal of ESCs but are not essential for pluripotency (Takahashi et al., 2003; Ogawa et al., 2007). For example, interference with the *Nodal* signaling has a strong effect on the self-renewal and proliferation of ESCs, but there is little effect on their pluripotency (Ogawa et al., 2007). The loss of *Eras* has a similar effect on the properties of ESCs (Takahashi et al., 2003).

The second cluster of genes is downregulated during ICM outgrowth, such as *Gata3*, *Gata4*, *Gata6*, *Cdx1*, *Cdx2*, *Pramel5*, *Pramel6*, *Pramel7*, *Sox17*, *Bmp15*, *Dppa1*, *Tbx3*, *Tbx20*, *Gdf9*, *Hoxd8*, *Gsc*, and *Klf17*. Some of these are developmental genes, including *Cdx2* and *Gata6*, which are required for development of the extraembryonic cells, trophectoderm and primary endoderm, respectively (Ralston and Rossant, 2008; Johnson et al., 2006; Koutsourakis et al., 1999), but they are apparently not essential for the self-renewal of ESCs (Figure S6B).

The third cluster of genes is highly expressed during ICM outgrowth, except in those neighboring cells that cease to be pluripotent as judged by the loss of expression of *Oct4*, *Sox2* and *Nanog*, as well as *Esrrb*, *Cdh1* (also known as *E-cadherin*), *Pecam1*, *Pim1*, *Pim3*, *Notch1*, *Notch4*, *Fzd9*, *Frz10*, *Dazl*, *Prdm14*, *Bmp8b*, and *Dppa4*, demonstrating a positive correlation with the pluripotency of ICM and ESCs (Figure S6C). Indeed, *Esrrb*, *E-cadherin*, *Pim1*, *Pim3*, and *Prdm14* have been shown to be important for pluripotency of ESCs (Ivanova et al., 2006; Chou et al., 2008; Aksoy et al., 2007; Tsuneyoshi et al., 2008).

The fourth cluster of genes is confined to cells that have ceased to be pluripotent as evident by the loss of expression of the Oct4-ΔPE-GFP reporter. This cluster of genes include *Hoxd8*, *Bmp1*, *Bmp2*, *Tgfbr2*, *Tgfbr3*, *Jak2*, *Fgf3*, *Fgf10*, *Fgfr3*, *Fgfr4*, *Sox7*, *Sox9*, *Sox17*, *Nanos1*, *Cdh5* (also known as *VE-cadherin*), and *Nkx6.2*, showing a negative correlation with the pluripotency of ESCs (Figure S6D). This cluster represents the earliest set of differentiation genes that are activated when the cells have just ceased to be pluripotent. For example, Fgf signaling has been shown to promote differentiation of ESCs (Kunath et al., 2007; Stavridis et al., 2007). Similarly, *Sox7* and *Sox17* also drive ESC differentiation (Séguin et al., 2008).

We also examined expression of primordial germ cell-specific genes during the course of blastocyst outgrowths (Zwaka and Thomson, 2005). We looked at the expression dynamics of early PGC markers during this process (Figure 3B; Table S2). We detected upregulation of *Ifitm3* (also known as *Fragilis*), *Prdm14*, *Ddx4* (also known as *Vasa*) and *Nanos3*, but *Dppa3* (also known as *stella*) was downregulated. We also found upregulation of *Prdm1* (also known as *Blimp1*), but later in ESCs, and the levels were highly variable in individual cells (Table S2). However, all these genes were repressed in the Oct4 negative outgrowth cells that had lost pluripotency. Further studies are required to assess the significance of these observations, if any, for the derivation and properties of ESCs.

### Functional Downstream Genes of Pluripotency Master Genes

The RNA-Seq analysis we have carried out provides an opportunity to identify pluripotency genes and their downstream targets, by comparing neighboring cells that are pluripotent to those that show a loss of pluripotency under the same culture conditions. These gene sets can also be compared with the expression of the earliest genes that are downregulated when ESCs are induced to differentiate in response to retinoic acid (RA) (Ivanova et al., 2006). We found a good correlation between our data and that reported previously with respect to 200 genes that were downregulated in both instances (Figure S6E). It is likely that our assay captured the earliest responding pluripotency genes, because we compared pluripotent cells and the corresponding earliest differentiated cells cultured under the same conditions.

Next, we asked if it is possible to determine the downstream targets of key pluripotency genes by analyzing genes that were downregulated in the day 5 outgrowth that had just ceased to be pluripotent. We compared our data with the reported effects of knockdown of *Nanog*, *Sox2*, *Oct4*, or *Esrrb*—genes described previously (Ivanova et al., 2006). We found clear overrepresentation of downregulated genes detected following loss of *Nanog* (p = 2.2 × 10^−16^), *Oct4* (p = 2.2 × 10^−16^), *Sox2* (p = 2.2 × 10^−16^), or *Esrrb* (p = 1.6 × 10^−7^) (Figure S6F). Furthermore, there was also strong overrepresentation of upregulated genes following knockdown of *Nanog* (p = 2.0 × 10^−8^), *Oct4* (p = 2.5 × 10^−10^), or *Sox2* (p = 2.2 × 10^−16^) (Figure S6G). This shows that our analysis serves as a good indicator of targets of pluripotency genes. Moreover, detection of these probable target genes that are regulated by corresponding pluripotency genes is cell-autonomous as seen by RNA-Seq within an individual cell, which excludes possible variable responses in individual cells analyzed in bulk cultures.

**Figure 6:**
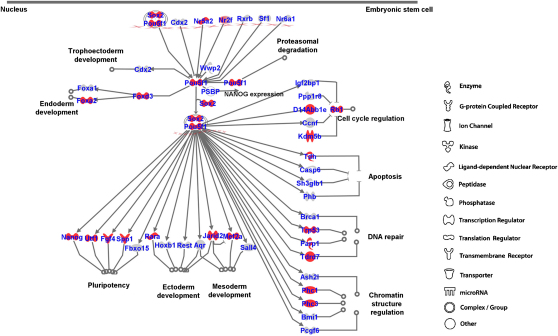
Gene Network Analysis of Oct4 in Embryonic Stem Cell Pluripotency Pathway The 22 genes (including *Oct4*) up/downregulated for more than 4-fold when the pluripotent cells lose pluripotency (FC[day 5 Oct4^+^/day 5 Oct4^−^] > 4 or < 0.25, p < 0.01) were shown in red. The gray colored genes have FC[day 5 Oct4^+^/day 5 Oct4^−^] < 4 and FC[day 5 Oct4^+^/day 5 Oct4^−^] > 0.25 (Table S4). The p value was estimated using Ingenuity systems software (http://www.ingenuity.com). FC, fold change.

To gain insight into the gene network underlying pluripotency, we aligned our data to the known gene network for Oct4 associated with embryonic stem cell pluripotency (Loh et al., 2006, 2008; Sharov et al., 2008). We found that this network is enriched in genes whose expression changed as judged by the comparison of pluripotent and neighboring nonpluripotent cells present within day 5 outgrowths. In fact, 22 out of the 45 Oct4 network genes changed their expression significantly by more than 4-fold. Of these 22 genes, only two genes, namely *Rb1* and *Foxa2*, were not directly regulated by *Oct4* (Figure 6; Table S4). This suggests that through single-cell digital transcriptome approach, we captured a large proportion of genes that are directly regulated by Oct4 in the network (p = 4.02 × 10^−6^) (Loh et al., 2006; Sharov et al., 2008). To our knowledge, this is the first time that the Oct4 gene network is validated in an individual cell, proving the cell-autonomous regulation of the network, which is an essential prerequisite for direct interactions within the gene network. Furthermore, we also aligned our data to the known gene network for human embryonic stem cell pluripotency and found a similar enrichment pattern. Forty-five out of the 137 human ESC network genes significantly changed their expression (p = 2.18 × 10^−4^, Figure S5C), suggesting conservation of the core aspects of the gene network of pluripotency between mouse and human, despite some known differences between them.

### Segregation of MicroRNAs Repressing Early Differentiation and Those Repressing Pluripotency

**Figure 7:**
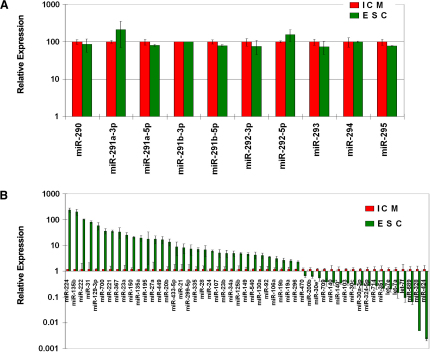
MicroRNA Expression in ICM and ESCs (A) miR-290 ∼−295 cluster microRNA expression in ICM and ESCs. (B) microRNAs showing significant differential expression between ICM and ESCs (p value < 0.01). The relative expression levels were shown (Table S5). The error bar represents the standard deviation calculated from three biological replicates.

We next wanted to see if microRNAs (miRNAs) have a role in the ICM outgrowths as they develop toward ESCs (Zamore and Haley, 2005; Gangaraju and Lin, 2009). Expression analysis of 330 miRNAs showed similar expression of pluripotency related miR-290 to -295 cluster in ICM and ESCs (Table S5; Figure 7A), while a set of 51 out of 330 miRNAs showed differential expression (Figure 7B). We found that let-7a, let-7e, let-7f, and let-7g were reduced between 4- and 12-fold in ESCs compared to ICM, which is reflected in the reduced levels of let-7 expression required for the self-renewal and maintenance of undifferentiated cancer stem cells (Yu et al., 2007). Correspondingly, we found 5-fold upregulation of *Lin28*, which is the suppressor of let-7 family miRNAs (Viswanathan et al., 2008). Recently, Card et al. (2008) reported that miR-302 cluster in human ESC is actively regulated by *Oct4* and *Sox2*, while miR-302a targets cell-cycle regulators and promotes an ESC-like cell cycle. Our miRNA profiling indicated that two members of miR-302 cluster, miR-302c and miR-367, increased 5- and 33-fold, respectively, from ICM to ESCs, which may contribute to the capacity for self-renewal of ESCs.

To explore the potential roles of miRNAs in regulating ESCs, we used the target prediction algorithms of PicTar, Miranda, or TargetScan (Krek et al., 2005; Lewis et al., 2005; Enright et al., 2003). To reduce possible “prediction noise,” we considered only those targets that are predicted by at least two algorithms. For the miRNAs highly expressed in both ICM and ESCs, there are two classes: the first class of miRNAs preferably target early differentiation genes (FC[Day5 Oct4^+^/Oct4^−^] < 0.25, r < −0.6, Table S6 and S7) (miR-19b, -19a, -106a, -20b, -106b, -9, -103, -107, -124a, -145; the target enrichment is 2.0-fold, p < 3.8 × 10^−6^). As a control, the targets of these ten miRNAs show no enrichment (p > 0.02) for pluripotency-related genes (FC[Day5 Oct4^+^/Oct4^−^] > 4, r > 0.6). The loss of this class of miRNAs may contribute to the phenotype of loss of pluripotent Oct4-positive epiblast cells when Dicer is knocked out in early embryos (Bernstein et al., 2003). The second class of miRNAs preferably target the ESC-specific pluripotency genes (FC[ESC/ICM] > 4) (miR-669b, -298, -692, -204, -28, -149, -34a, -182, ↑-129-5p, -133a, -320; the target enrichment is 1.6-fold, p < 1.3 × 10^−8^). As a control, the targets of these eleven miRNAs show no enrichment (p > 0.03) in ICM-specific genes (FC[ESC/ICM] < 0.25). The loss of this class of miRNAs may contribute to the phenotype of resistance to differentiation when Dicer or DGCR8, two key components of the miRNA processing pathway, are knocked out in established ESCs (Kanellopoulou et al., 2005; Wang et al., 2007). Taken together, miRNAs may contribute to ESC's ability to maintain the balance between pluripotency and the potential for rapid differentiation, through one set of miRNAs targeting genes that drive differentiation, while a separate set of miRNAs target ESC-specific pluripotency genes.

### Conclusion

Our study provides insight into the dynamic molecular changes that accompany cell-fate changes. During the conversion of ICM cells to ESCs, there is an evident arrest of a normal developmental program, which is subverted in vitro in favor of a potential for unrestricted self-renewal while retaining the ability to undergo differentiation into all the diverse cell types. We demonstrate how both the retention of expression of key genes allows inheritance of a fundamental property of the ICM, namely pluripotency, while other changes in the transcriptome permit exit from a normal developmental program and confer a key property of self-renewal. Changes in epigenetic regulators apparently allow for the stability of the newly acquired epigenotype, which is crucial for the inherent plasticity of ESCs. The conversion from ICM to ESC is also coupled with a role for distinct sets of miRNAs that allow for both self-renewal while the cells retain the ability to respond rapidly to cues for differentiation. Our investigation may serve as a paradigm for other studies, including regulation and differentiation of small numbers of stem cells in adults. Our approach is applicable to studies on small groups of differentiating cells and for gaining insight into how developmental programs might be undermined, leading to the formation of diseased tissues, including cancers.

## Experimental Procedures

### Isolation of Embryos and Single Cells

All embryos were recovered from 129 females mated with Oct4-ΔPE-GFP transgenic male mice. The transgenic GFP expression of the reporter is under the control of Oct4 promoter and distal enhancer, but the proximal enhancer region is deleted. This GFP transgene reporter shows expression in the E3.5 ICM and E4.5 Epiblast of blastocysts and PGC in vivo and in ESC (Yeom et al., 1996). E3.5 and E4.5 blastocysts were flushed from the uterus of 129 pregnant females. For ESC outgrowth, E3.5 blastocysts were cultured in KSOM medium for the first day and then transferred to GMEM medium (GIBCO, cat. no. 21710-025) with 15% Fetal Calf Serum (FCS) (GIBCO, cat. no. 16000-044) and 1000 U/ml Lif on mitomycin C-treated MEF feeder cells for all later periods. The time when the E3.5 blastocysts were placed into culture was designated as day 0.

For the isolation of single cells of E3.5 ICM or E4.5 epiblast, the blastocysts were first placed in a mouse trophoblast antibody for 30 min. Then they were treated by complement for 30 min. After this, the lysed trophectoderm cells were removed and the isolated ICM or epiblast was placed in EGTA-PBS for 10 min. After that, they were furthered treated by Trypsin at 37°C for 5 min. Then they were transferred into GMEM medium with 15% FCS and dissociated into single-cell suspension. The resulting single cells were washed in BSA-PBS twice and prepared to be picked as single cells.

For the isolation of blastocyst/ICM outgrowth, it was treated by trypsin for 5 min to dissociate the core part of outgrowth from surrounding trophectoderm progenies. The inner core of cells in the outgrowth was treated with EGTA-PBS for 10 min at room temperature and trypsin for 5 min at 37°C. The core of cells was dissociated by pipetting into a single-cell suspension in GMEM medium with 15% FCS. Next, the GFP-positive and -negative cells were separated manually under a fluorescence microscope. The single cells were washed in BSA-PBS twice before they were picked individually for subsequent analysis.

### Preparation of Single-Cell cDNAs

The single-cell RNA-seq method has been described in detail previously (Tang et al., 2009, 2010). In brief, an individual cell was manually picked and transferred into lysate buffer by a mouth pipette, followed by reverse transcription directly on the whole-cell lysate. Following this procedure, terminal deoxynucleotidyl transferase was used to add a poly(A) tail to the 3′ end of first-strand cDNAs, which was followed by 20 + 9 cycles of PCR to amplify the single-cell cDNAs.

### RNA-Seq Library Preparation, Sequencing, and Alignment

After generation of the target cDNA from a single cell, 100 ng cDNA (0.5–3 kb) was sheared into 80–130 bp fragments. P1 and P2 adaptors were ligated to each end, and the fragments were subjected to 8–10 cycles of PCR amplification. Emulsion PCR reactions were performed by combining 1.6 billion 1 μm diameter beads that had P1 primers covalently attached to their surfaces with 500 pg of single-cell libraries. Applied Biosystems SOLiD sequencer generated 50-base sequences, and AB's whole transcriptome software tools were used to analyze the sequencing reads (http://solidsoftwaretools.com/gf/project/transcriptome/). The reads obtained from each cell were matched to the Mouse genome (mm 9, NCBI Build 37) and reads that aligned uniquely were used in the downstream analysis. These reads were used to create base coverage files (in a wiggle format), which can be viewed directly in the UCSC genome browser, or to detect known or novel exon-exon junctions. Unambiguously mapped reads were first used to generate exon counts and then transcript or gene counts. Feature counts were normalized using the RPM (read per million aligned reads) method, and no adjustment to gene/transcript size was made because our protocol has a limited coverage of 0.5–3 kb from the 3′ end of the transcripts. An alternative analysis was used for alignments that were not aligned to their full length, where reads were aligned to a reference containing exon-exon junctions, using 42 bases on each side for junctions, allowing up to four mismatches for the full length of the read (50 bases) (Tang et al., 2009). The quality of the single-cell RNA-Seq data was analyzed (Figure S7). These analyses showed that our single-cell RNA-Seq data are highly reproducible, reliable, and accurate for ICM outgrowth and ESCs.

### Real-Time PCR

For TaqMan real-time PCR, 1.0 μl of diluted cDNAs was used for each 10 μl real-time PCR (1× PCR Universal Master Mix, 250 nM TaqMan probe, 900 nM of each primer, that are commercially available as ready to use Assays, custom-plated in 384-plates or TaqMan low Density Array cards by Applied Biosystems). All reactions were duplicated. The PCR was done as following using an AB7900 with 384-well plates: first, 95°C for 10 min to activate the Taq polymerase, then 40 cycles of 95°C for 15 sec and 60°C for 1 min.

### MicroRNA Profiling of ICM and ESCs

The detailed protocol is described previously (Tang et al., 2006). In brief, 10 cells were picked into a PCR tube by glass capillary and were lysed by heat treatment at 95°C for 5 min. Then the microRNAs were reverse transcribed into cDNAs by pool of 330 of stem-looped primers. After this, these microRNA cDNAs were amplified by 18 cycles of PCR by 330 forward primers and a universal reverse primer. Finally the cDNAs were split and each individual microRNA was measured by TaqMan probe-directed real-time PCR. Three biological replicates were done for each type of cell.

## Accession Numbers

All of the raw data of single-cell RNA-Seq for ICM outgrowth have been deposited in the high-throughput sequencing database of GEO (accession number GSE20187).

## Supplemental Information

Document S1. Seven Figures

Table S1. The expression of 385 ESC-Related Genes during ICM Outgrowth by Single-Cell Real-Time PCR

Table S2. Single-Cell RNA-Seq RefSeq Transcripts and Splice Variants Counts for ICM Outgrowth Single-cell RNA-Seq counts (1) mapped to RefSeq for ICM, ESC and ICM outgrowth; (2) mapped to unique known exon-exon junctions and exons for each known splicing variant in RefSeq.

Table S3. Summary of RNA-Seq Alignments for ICM, ESC, and ICM Outgrowth

Table S4. GO Analysis of Functions, Networks, and Pathways for ICM Outgrowth GO analysis results of functions, networks, and pathways for (1) Refseq transcripts with FC(ESC/ICM) > 4 or FC (ESC/ICM) < 0.25, p < 0.01; (2) Refseq transcripts with FC[Day 5-Oct4^+^/Day5-Oct4^−^]>4 or FC[Day5-Oct4^+^/Day5-Oct4^−^]<0.25, p<0.01); (3) splicing variances with FC[ESC/ICM, junctions]>2 or FC[ESC/ICM, junctions]<0.5, p<0.01; (4) genes involved in Oct4 pathways; and (5) genes involved in stem cell pluripotency pathways using Ingenuity systems software (http://www.ingenuity.com); (6) Expression changes of 114 epigenetic regulators between ICM and ESCs

Table S5. MicroRNA Expression Profile of ICM from E3.5 Blastocysts and ESCs

Table S6. MicroRNA Enrichment Calculations Using Target Prediction Algorithms of PicTar, Miranda, or TargetScan

Table S7. The Lists of 1,378 Potential Pluripotency Genes The lists of 1,378 potential pluripotency genes (FC[Day5-Oct4^+^/Day5-Oct4^−^]>4, p<0.01, and r >0.6). Here r represents the averaged correlation coefficient with the expression of *Oct4*, *Sox2*, and *Nanog* in ICM outgrowth.
