# Supplementary material for: Tracing the Derivation of Embryonic Stem Cells from the Inner Cell Mass by Single-Cell RNA-Seq Analysis
Source: Cell Stem Cell. 2010 May 7;6(5-2):468–78. doi: 10.1016/j.stem.2010.03.015 (PMC2954317; doi:10.1016/j.stem.2010.03.015)
Supplement: Document S1. Seven Figures [file mmc1.pdf]

Cell Stem Cell, Volume *6*

Supplemental Information

Tracing the Derivation of Embryonic Stem Cells from the Inner Cell Mass by Single-Cell RNA-Seq Analysis

Fuchou Tang, Catalin Barbacioru, Siqin Bao, Caroline Lee, Ellen Nordman, Xiaohui Wang, Kaiqin Lao, and M. Azim Surani

Supplemental Inventory

Items	Related to	General description
Figure S1	Figure 2	Gene expression dynamics measured by real-time PCR in single ICM outgrowth cells. The heterogeneity of ICM outgrowth, and the intermediate states of cells when they lose pluripotency were shown at single cell resolution.
Figure S2	Figure 3	Correlation plots and Pearson correlation coefficients of RNA-Seq data of single ICM outgrowth cells. The gene expression dynamics during ICM outgrowth at whole genome scale were shown.
Figure S3	Figure 3	Gene expression pattern between ICM, epiblast, and ESCs. The general overlap of the genes expressed between these types of cells was shown.
Figure S4	Figure 5	Splice specific differential expression of Dppa4 in ICM, epiblast, and ESCs. The dramatic regulation of alternative splicing was shown at single base resolution.
Figure S5	Figure 6	Gene network analysis of different pathways. Several of the gene networks were validated within an individual cell.
Figure S6	Figure 3	Gene expression dynamics during ICM outgrowth. The expression pattern of the genes related to pluripotency and self-renewal ability was shown.
Figure S7	Figure 3	Quality check of the single cell RNA-Seq data for ICM outgrowth. These analyses showed that the single cell RNA-Seq data are highly reproducible, reliable, and accurate for ICM outgrowth and ESCs.
Table S1	Figure 1 & Figure 2	The expression of 385 ESC related genes during ICM outgrowth by single cell real-time PCR.
Table S2	Figure 3, Figure 4, Figure 5, & Figure 6	Single cell RNA-Seq RefSeq transcripts and splice variants counts for ICM outgrowth.
Table S3	Figure 3	Summary of RNA-Seq alignments for ICM outgrowth.
Table S4	Figure 6	GO analysis of functions, networks, and pathways for ICM outgrowth.
Table S5	Figure 7	MicroRNA expression profile of ICM from E3.5 blastocysts and ESCs.
Table S6	Figure 7	MicroRNA enrichment calculations using target prediction algorithms of PicTar, Miranda, or TargetScan.
Table S7	Figure 3 & Figure 7	The lists of 1,378 potential pluripotency genes.

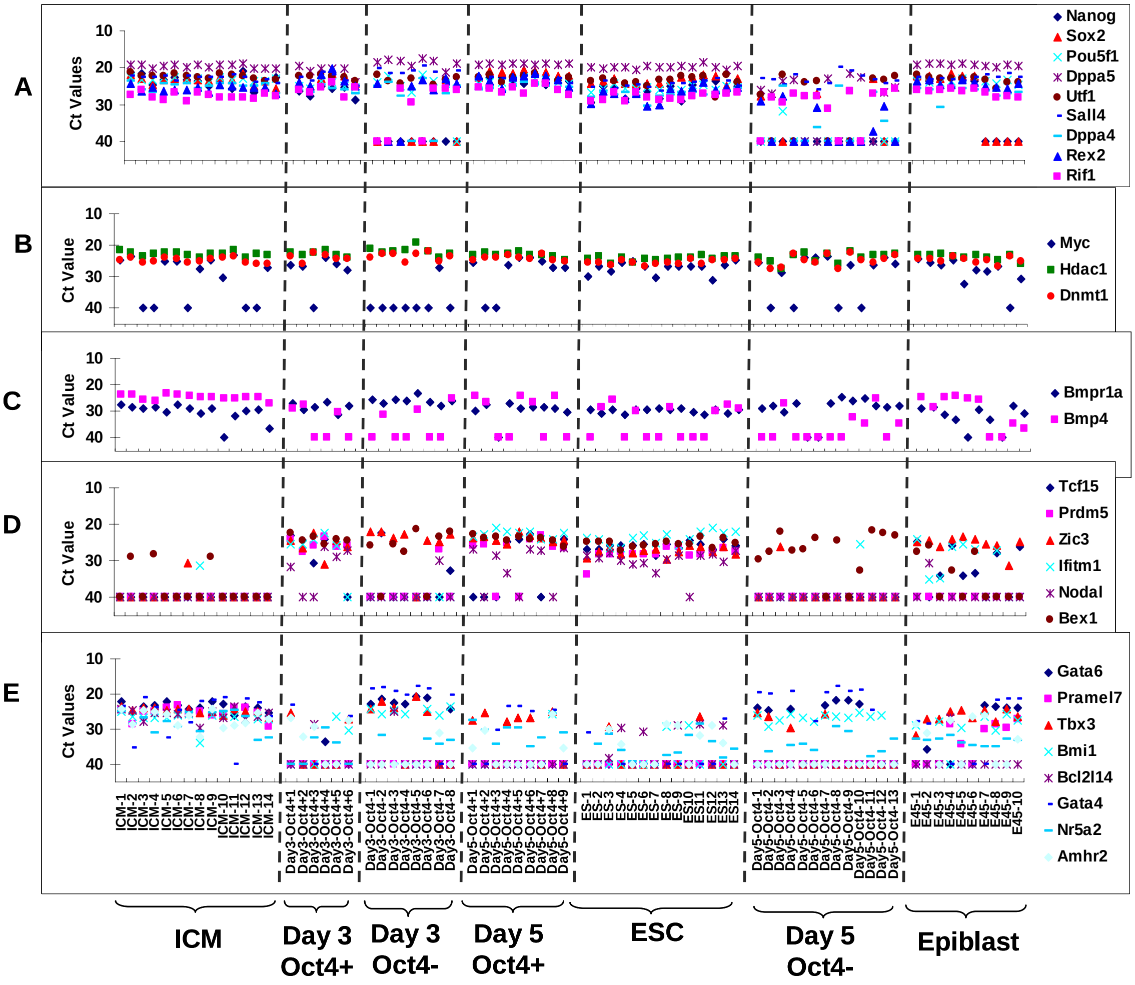
A
B
C
D
E
Ct Values
Ct Value
10
20
30
40
Nanog
Sox2
Pou5f1
Dppa5
Utf1
Sall4
Dppa4
Rex2
Rif1
Myc
Hdac1
Dnmt1
Bmpr1a
Bmp4
Tcf15
Prdm5
Zic3
Ifitm1
Nodal
Bex1
Gata6
Pramel7
Tbx3
Bmi1
Bcl2l14
Gata4
Nr5a2
Amhr2
ICM-1
ICM-2
ICM-3
ICM-4
ICM-5
ICM-6
ICM-7
ICM-8
ICM-9
ICM-10
ICM-11
ICM-12
ICM-13
ICM-14
Day3-Oct4+1
Day3-Oct4+2
Day3-Oct4+3
Day3-Oct4+4
Day3-Oct4+5
Day3-Oct4+6
Day3-Oct4-1
Day3-Oct4-2
Day3-Oct4-3
Day3-Oct4-4
Day3-Oct4-5
Day3-Oct4-6
Day3-Oct4-7
Day3-Oct4-8
Day5-Oct4+1
Day5-Oct4+2
Day5-Oct4+3
Day5-Oct4+4
Day5-Oct4+5
Day5-Oct4+6
Day5-Oct4+7
Day5-Oct4+8
Day5-Oct4+9
ES-1
ES-2
ES-3
ES-4
ES-5
ES-6
ES-7
ES-8
ES-9
ES10
ES11
ES12
ES13
ES14
Day5-Oct4-1
Day5-Oct4-2
Day5-Oct4-3
Day5-Oct4-4
Day5-Oct4-5
Day5-Oct4-6
Day5-Oct4-7
Day5-Oct4-8
Day5-Oct4-9
Day5-Oct4-10
Day5-Oct4-11
Day5-Oct4-12
Day5-Oct4-13
E45-1
E45-2
E45-3
E45-4
E45-5
E45-6
E45-7
E45-8
E45-9
E45-10
ICM
Day 3 Oct4+
Day 3 Oct4-
Day 5 Oct4+
ESC
Day 5 Oct4-
Epiblast

Figure S1

Figure S1 Gene expression dynamics measured by Real-time PCR in single cells of fourteen ICM cells (E3.5), ten epiblast cells (E4.5), six Day3 Oct4 positive ICM outgrowth cells, eight Day3 Oct4 negative ICM outgrowth cells, nine Day5 Oct4 positive ICM outgrowth cells, thirteen Day5 Oct4 negative ICM outgrowth cells, and fourteen ESCs: (A) *Oct4, Sox2, Nanog, Dppa4, Dppa5, Sall4, Utf1, Rex2*, and *Rif1* (B) *Myc, Dnmt1*, and *Hdac1*; (C) *Bmp4* and *Bmpr1a*; (D) *Tcf15, Prdm5, Zic3, Ifitm1, Nodal,* and *Bex1*; (E) *Gata4, Gata6, Pramel7, Tbx3, Bmi1, Bcl2l14, Nr5a2*, and *Amhr2* (Table S1).

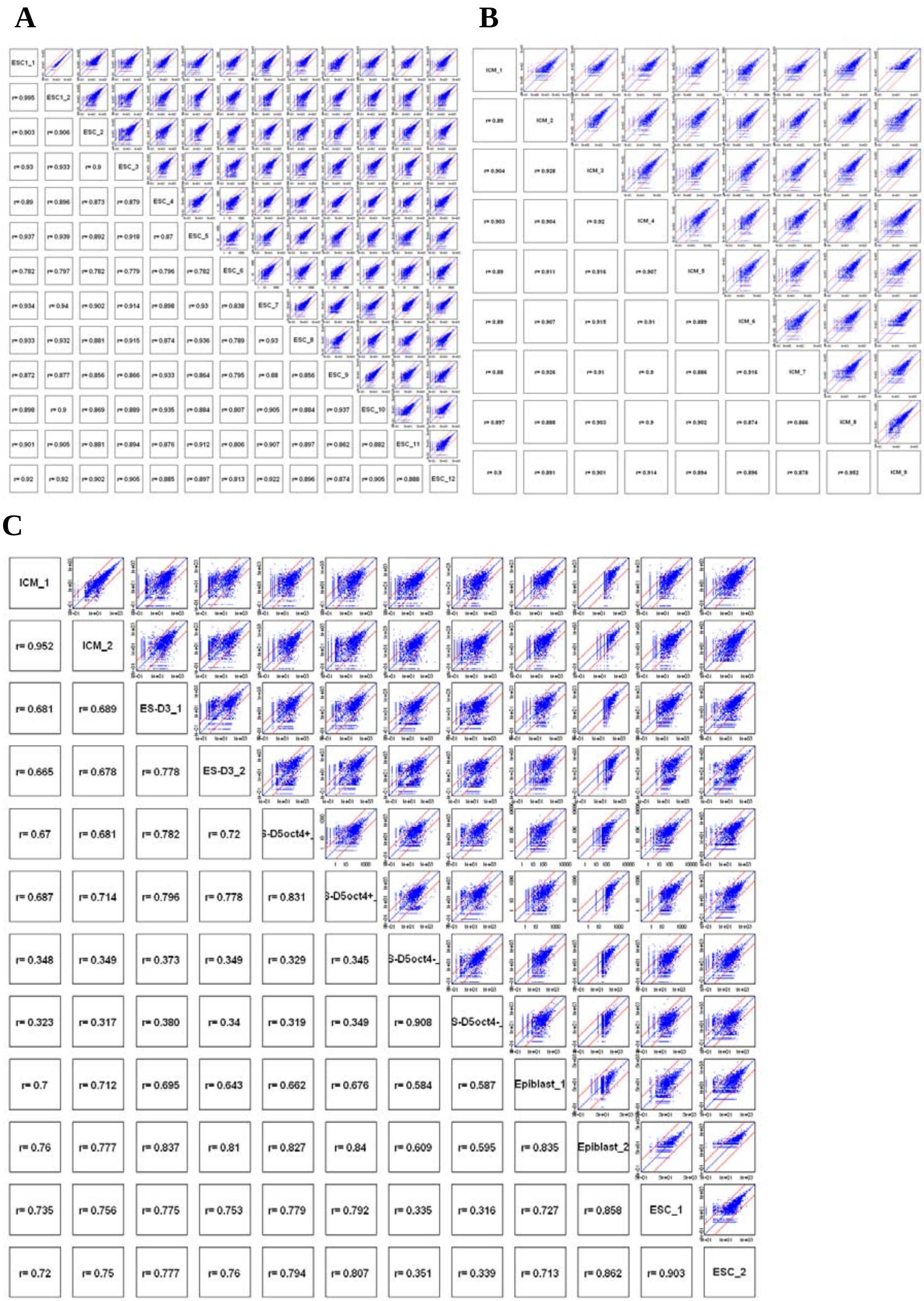
A
B
C
ESC1_1
ESC1_2
ESC_2
ESC_3
ESC_4
ESC_5
ESC_6
ESC_7
ESC_8
ESC_9
ESC_10
ESC_11
ESC_12
ICM_1
ICM_2
ICM_3
ICM_4
ICM_5
ICM_6
ICM_7
ICM_8
ICM_9
ICM_1
ICM_2
ES-D3_1
ES-D3_2
S-D5oct4+_
S-D5oct4+_
S-D5oct4-_
S-D5oct4-_
Epiblast_1
Epiblast_2
ESC_1
ESC_2
r= 0.952
r= 0.681
r= 0.689
r= 0.665
r= 0.678
r= 0.778
r= 0.67
r= 0.681
r= 0.782
r= 0.72
r= 0.687
r= 0.714
r= 0.796
r= 0.778
r= 0.831
r= 0.348
r= 0.349
r= 0.373
r= 0.349
r= 0.329
r= 0.345
r= 0.323
r= 0.317
r= 0.380
r= 0.34
r= 0.319
r= 0.349
r= 0.908
r= 0.7
r= 0.712
r= 0.695
r= 0.643
r= 0.662
r= 0.676
r= 0.584
r= 0.587
r= 0.76
r= 0.777
r= 0.837
r= 0.81
r= 0.827
r= 0.84
r= 0.609
r= 0.595
r= 0.835
r= 0.735
r= 0.756
r= 0.775
r= 0.753
r= 0.779
r= 0.792
r= 0.335
r= 0.316
r= 0.727
r= 0.858
r= 0.72
r= 0.75
r= 0.777
r= 0.76
r= 0.794
r= 0.807
r= 0.351
r= 0.339
r= 0.713
r= 0.862
r= 0.903

Figure S2

Figure S2 Correlation plots and Pearson correlation coefficients of (A) 12 single ESCs, (B) 9 single ICM cells, and (C) 12 representative ICM outgrowth cells (Table S2).

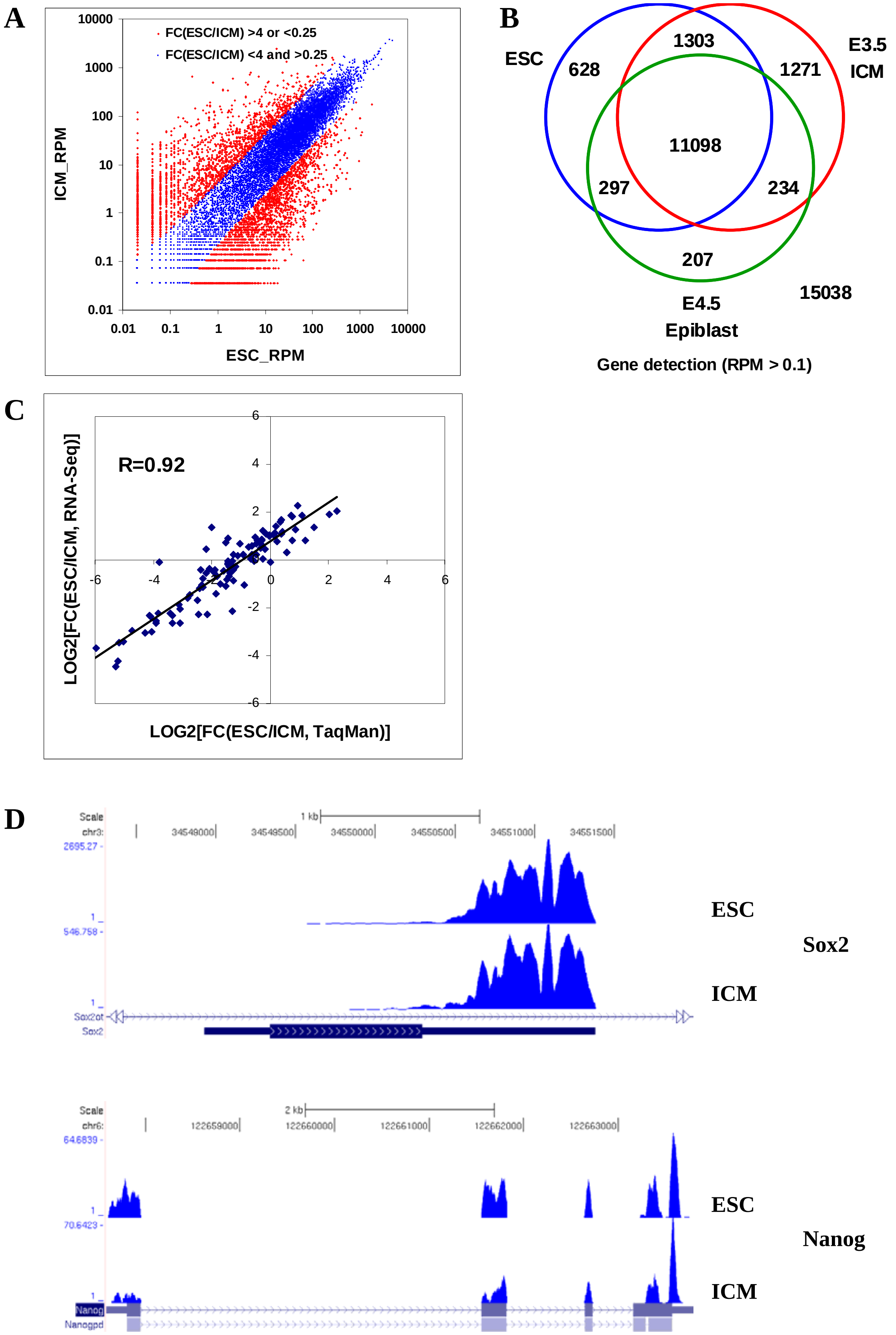
A
10000
1000
100
10
1
0.1
0.01
ICM_RPM
FC(ESC/ICM) >4 or <0.25
FC(ESC/ICM) <4 and >0.25
0.01
0.1
1
10
100
1000
10000
ESC_RPM
B
ESC
628
1303
1271
E3.5
ICM
11098
297
234
207
15038
E4.5
Epiblast
Gene detection (RPM > 0.1)
C
R=0.92
LOG2[FC(ESC/ICM, RNA-Seq)]
LOG2[FC(ESC/ICM, TaqMan)]
D
ESC
ICM
Sox2
Sox2ot
ESC
ICM
Nanog
Nanogpd

Figure S3

Figure S3 Gene expression pattern between ICM, epiblast, and ESCs. (A) Correlation plots for ESC vs ICM. The blue and red dots are, respectively, genes with fold changes less than or greater than 4; (B) Venn diagram of the genes expressed in ESC, E3.5 ICM, and E4.5 Epiblast (Table S2); (C) The correlation plot of the fold changes that are determined by RNA-Seq, LOG2 FC[ESC/ICM], and TaqMan real-time PCR, LOG2 of FC[ESC/ICM]; (D) Cover plots of Sox2 and Nanog in ESCs and ICM.

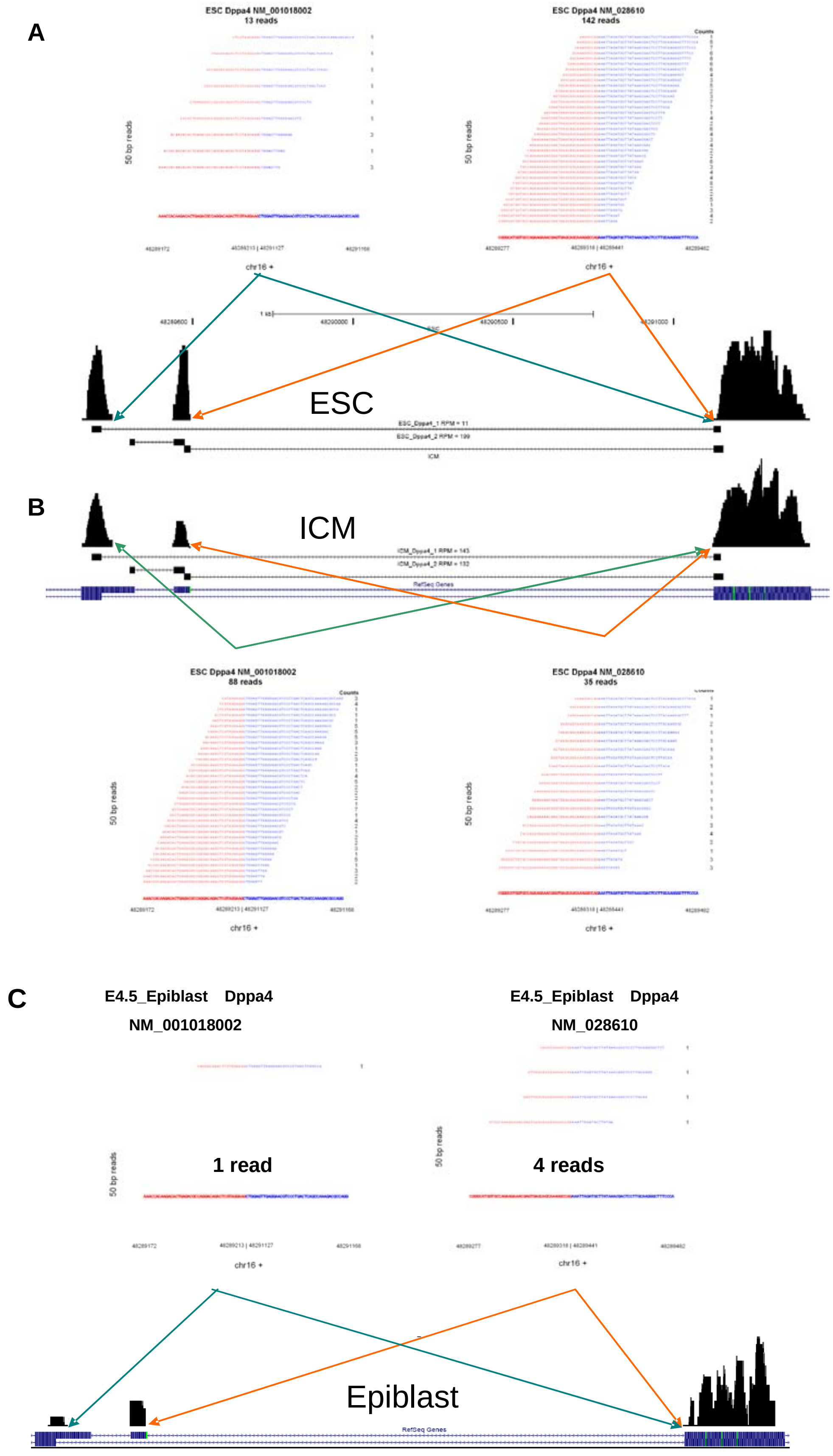
A
ESC Dppa4 NM_001018002
13 reads
ESC Dppa4 NM_028610
142 reads
50 bp reads
chr16 +
ESC
B
ICM
ESC Dppa4 NM_001018002
88 reads
ESC Dppa4 NM_028610
35 reads
C
E4.5_Epiblast Dppa4
NM_001018002
E4.5_Epiblast Dppa4
NM_028610
1 read
4 reads
Epiblast

Figure S4

Figure S4 Splice specific differential expression of Dppa4 in ICM, epiblast, and ESCs. RNA-Seq reads aligned to regions (exonic or exon-exon junctions) specific to a single transcript are used to differentiate between spliced transcripts. Dppa4 gene is known to encode two alternative spliced forms NM_001018002 (transcript variant #1, which has one specific exon and 2 specific exon-exon junctions, one of them being represented in this plot) and NM_028610 (transcript variant #2, which has one specific exon-exon junction). The number of reads covering NM_001018002 specific junction in (A) ESC (13 counts) is much less than the coverage obtained from (B) ICM cells (88 counts). The opposite trend is observed for NM_028610 specific exon-exon junction, for which we observe more reads in ESC (142 counts) compared to the reads observed the ICM (35 counts, Table S2).

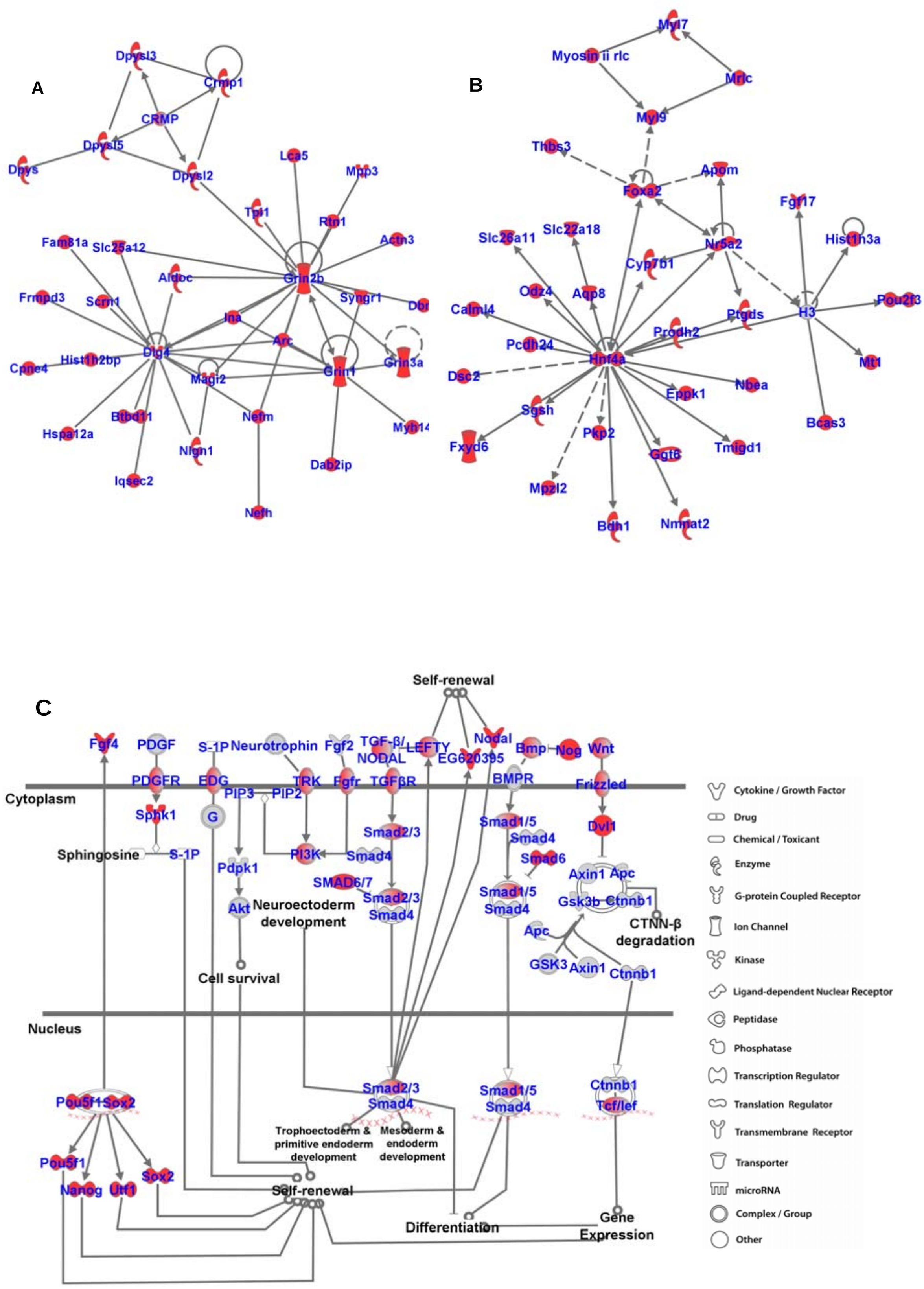
A
B
C
Self-renewal
Cytoplasm
Nucleus
Neuroectoderm development
Cell survival
CTNN-β degradation
Trophoectoderm & primitive endoderm development
Mesoderm & endoderm development
Differentiation
Gene Expression
Cytokine / Growth Factor
Drug
Chemical / Toxicant
Enzyme
G-protein Coupled Receptor
Ion Channel
Kinase
Ligand-dependent Nuclear Receptor
Peptidase
Phosphatase
Transcription Regulator
Translation Regulator
Transmembrane Receptor
Transporter
microRNA
Complex / Group
Other

Figure S5

Figure S5 Gene network analysis of different pathways. (A) Gene network plots of nucleic acid metabolism genes; (B) Gene network plots of lipid metabolism genes, which show dynamic changes of expression between ICM and ESC (FC[ESC/ICM]>4 or <0.25, p<0.01, Table S4). (C) Gene network plots of stem cell pluripotency pathway. 45 out of the 137 genes in this network are up/down regulated for more than 4 folds when the pluripotent cells lose pluripotency (The genes of FC[Day5-Oct4$^+$/Day5-Oct4$^-$]>4 or FC[Day5-Oct4$^+$/Day5-Oct4$^-$]<0.25, p<0.01 are shown in red color, Table S4). The p-value was estimated using Ingunity systems software (www.ingenuity.com).

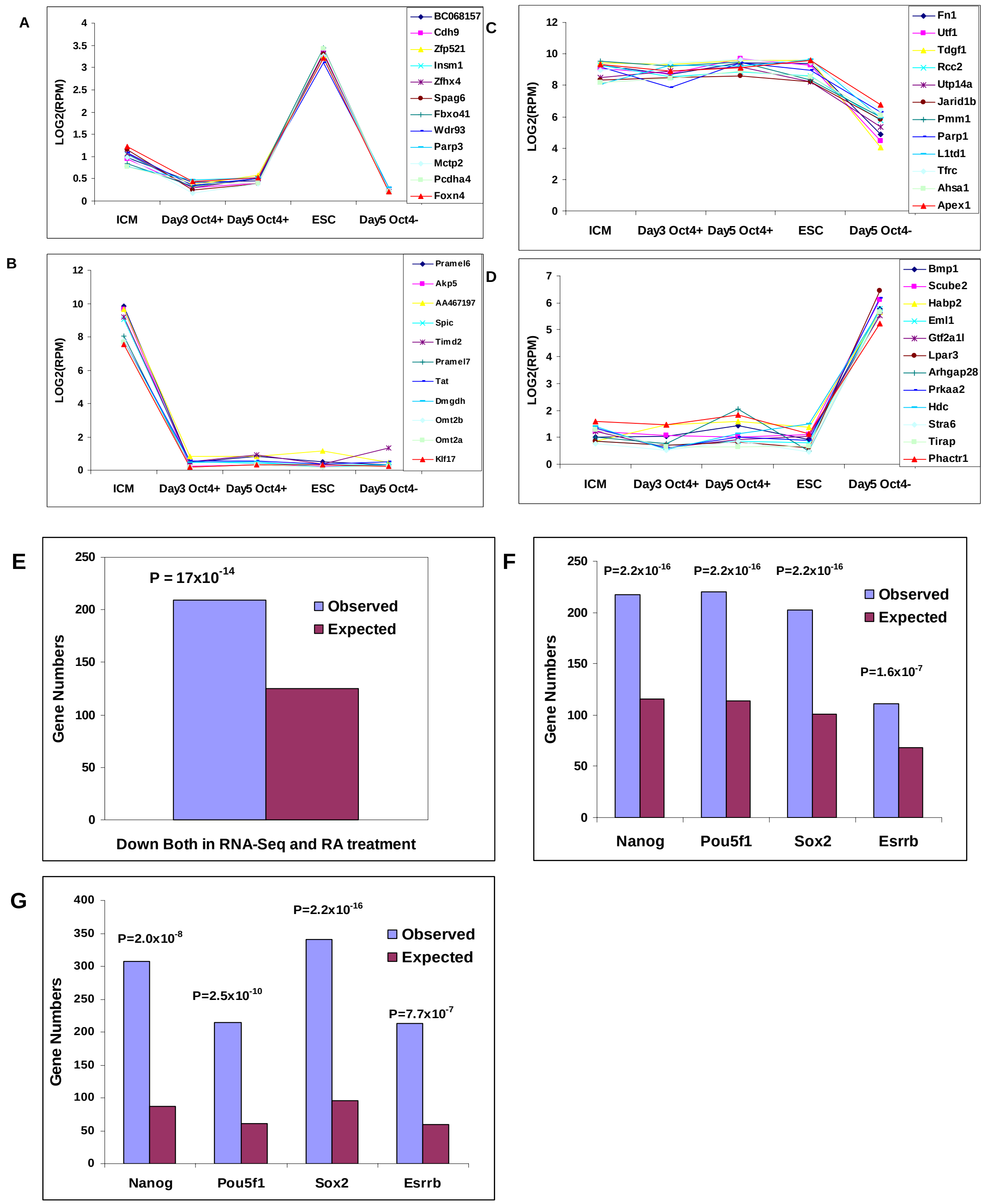
A
LOG2(RPM)
ICM
Day3 Oct4+
Day5 Oct4+
ESC
Day5 Oct4-
BC068157
Cdh9
Zfp521
Insm1
Zfhx4
Spag6
Fbxo41
Wdr93
Parp3
Mctp2
Pcdha4
Foxn4
B
Pramel6
Akp5
AA467197
Spic
Timd2
Pramel7
Tat
Dmgdh
Omt2b
Omt2a
Klf17
C
Fn1
Utf1
Tdgf1
Rcc2
Utp14a
Jarid1b
Pmm1
Parp1
L1td1
Tfrc
Ahsa1
Apex1
D
Bmp1
Scube2
Habp2
Eml1
Gtf2a1l
Lpar3
Arhgap28
Prkaa2
Hdc
Stra6
Tirap
Phactr1
E
Gene Numbers
P = 17x10^-14
Observed
Expected
Down Both in RNA-Seq and RA treatment
F
P=2.2x10^-16
P=2.2x10^-16
P=2.2x10^-16
P=1.6x10^-7
Nanog
Pou5f1
Sox2
Esrrb
G
P=2.0x10^-8
P=2.5x10^-10
P=2.2x10^-16
P=7.7x10^-7

Figure S6

Figure S6 Gene expression dynamics during ICM outgrowth. (A) - (D), Candidate genes related to pluripotency and self-renewal ability of ESCs. (A) Expression profiling of the representative 12 genes that have low expression in E3.5 ICM, Day3 $Oct4^+$, and Day5 $Oct4^+$ ICM outgrowth cells, but are upregulated significantly in ESCs; (B) Expression profiling of the representative 11 genes that have high expression in E3.5 ICM, then decrease significantly at Day3 $Oct4^+$, Day5 $Oct4^+$ and Day5 $Oct4^-$ ICM outgrowth cells. Here, the averaged intensities of three single cells were plotted. (C) Expression profiling of the representative 12 genes that have high expression in ICM, Day3 $Oct4^+$ and Day5 $Oct4^+$ ICM outgrowth cells, and ESCs, but are downregulated significantly in Day5 $Oct4^-$ ICM outgrowth cells with no pluripotency; (D) Expression profiling of the representative 12 genes that have low expression in ICM, Day3 $Oct4^+$ and Day5 $Oct4^+$ ICM outgrowth cells, and ESCs, but are upregulated by more than 4 folds in Day5 $Oct4^-$ ICM outgrowth cells with no pluripotency. The averaged intensities of three single cells were plotted here (Table S2). (E) - (F): Enrichment of downstream genes of pluripotency master genes. (E) The earliest downregulated genes when the differentiation of ESC is induced by all-trans retinoic acid (RA) are overrepresented in the downregulated genes when pluripotent cells lose pluripotency in our assay. (F) The functional downstream genes of *Nanog, Oct4, Sox2*, and *Esrrb* that are down regulated both after the knockdown of these genes and when pluripotent cells lose pluripotency. (G) The functional downstream genes of *Nanog, Oct4, Sox2*, and *Esrrb* that are up regulated both after the knockdown of these genes and when pluripotent cells lose pluripotency. The number of the expected genes is equal to the number of the genes up- or down- regulated when one of the pluripotent gene is knocked down (FC>1.5), divided by the total number of the genes detected by RNA-Seq (RPM>0.1), and multiplied by the number of the genes up- or down- regulated in Day5 outgrowth when the pluripotent cells lose pluripotency (FC >2 or <0.5).

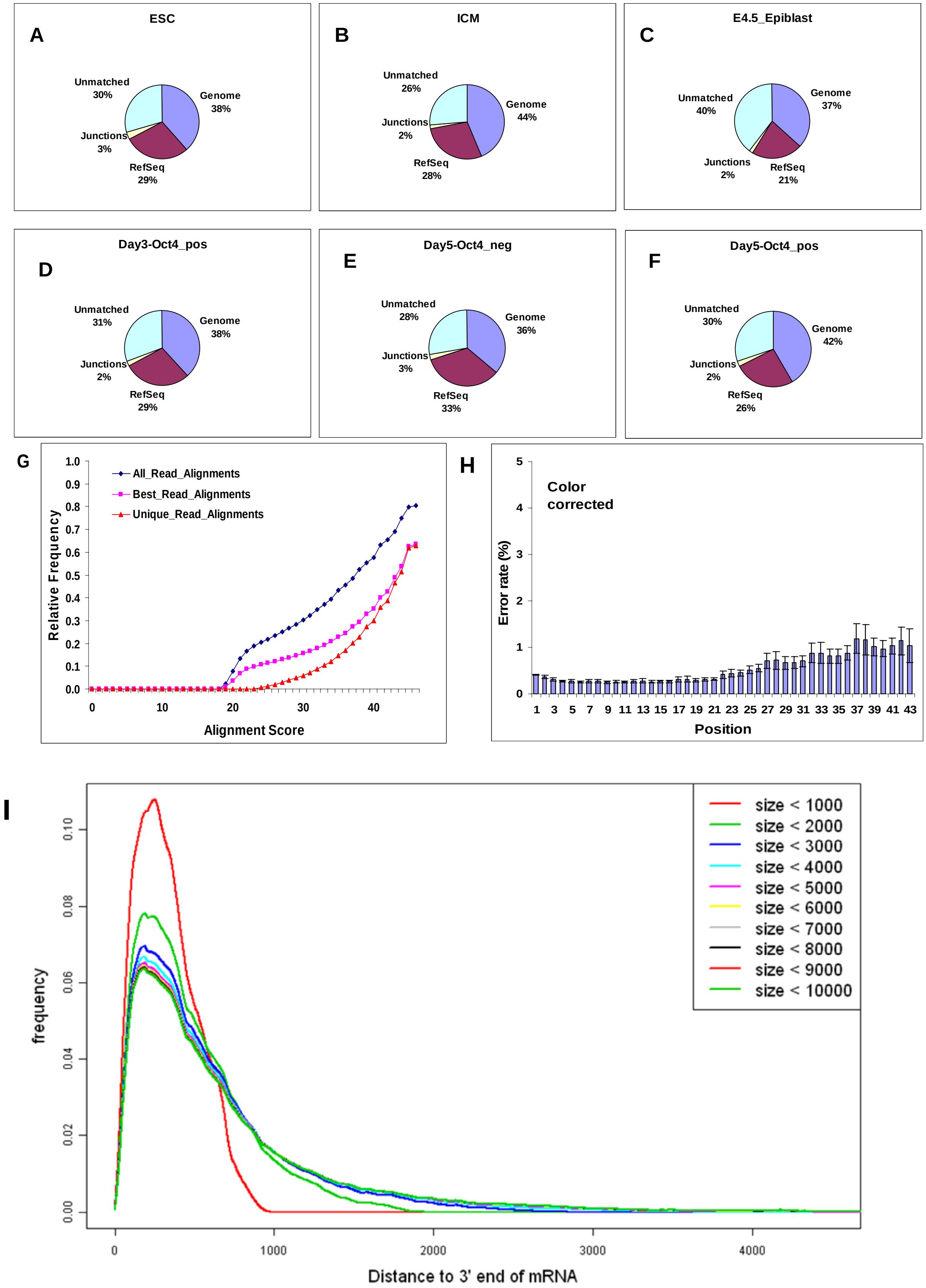
A
ESC
Unmatched 30%
Genome 38%
Junctions 3%
RefSeq 29%
B
ICM
Unmatched 26%
Genome 44%
Junctions 2%
RefSeq 28%
C
E4.5_Epiblast
Unmatched 40%
Genome 37%
Junctions 2%
RefSeq 21%
D
Day3-Oct4_pos
Unmatched 31%
Genome 38%
Junctions 2%
RefSeq 29%
E
Day5-Oct4_neg
Unmatched 28%
Genome 36%
Junctions 3%
RefSeq 33%
F
Day5-Oct4_pos
Unmatched 30%
Genome 42%
Junctions 2%
RefSeq 26%
G
All_Read_Alignments
Best_Read_Alignments
Unique_Read_Alignments
Relative Frequency
Alignment Score
H
Color corrected
Error rate (%)
Position
I
size < 1000
size < 2000
size < 3000
size < 4000
size < 5000
size < 6000
size < 7000
size < 8000
size < 9000
size < 10000
frequency
Distance to 3' end of mRNA

Figure S7

Figure S7 Quality check of the single cell RNA-Seq data for ICM outgrowth. (A) - (F): Pie charts of the number of RNA-Seq reads. Reads are mapped to the mouse genome (mm9, NCBI Build 37) as described by Tang et al (2009) in the Alignment and Algorithm section. We used UCSC annotation database (mm9) to determine if matching locations of individual reads correspond to exon regions, or exon-exon junctions of known transcripts. The number of these reads as a fraction of the total number of reads produced by cell type is represented in these pie charts. We generated 500 millions reads in total (Table S3). We obtained about 60 - 74% of reads that mapped uniquely to Refseq, known junctions, and the genome. There are about 2 - 3 % reads that mapped uniquely to known exon junctions. Here, Genome means these reads uniquely mapped to the mouse genome, but did not map to RefSeq transcripts. The percentages are calculated based on aggregated reads for each cell type. The detailed alignment summary for each individual cell is listed in Table S3. (G) Alignment score for RNA-Seq reads. For each read alignment we calculate the score obtained by adding 1 for a color match and subtracting 1 for a mismatch. This scoring function is used in the extension step (described by Tang et al (2009) in the Alignment and Algorithm section). Cumulative distributions of all alignment scores, best read alignment, and alignment scores of reads aligning to a unique location, from all ESC cells, are shown. Low scoring alignments are expected to be produced by reads aligning over splice junctions or low quality reads, while high scoring reads are expected to represent exonic regions. Here, we only used unique reads for our RefSeq transcripts and junctions mapping. (H) The estimated sequencing error rate of SOLiD system. Reads that aligned contiguously to the genome (as described in Alignment and Algorithm section), on full length and to a unique location are used to estimate instrument error rate as a function of position within the read (see Tang et al Error detection section). For a given position, the number of times we see a difference between the read call and corresponding genomic location, calculated as a fraction of all reads considered, is represented on the y-axis. Data produced from all ESC and ICM cells is aggregated and represented as average error rates, and 95% confidence intervals of these estimates are inferred. The averaged error rate is about 0.5% for the first 30 positions. For 50-mer reads, we only plotted the first 44 bases since the extension step reaches full length of the read if

reads have fewer errors on the last positions (and so the error rates of the last positions for the subset of reads we used are under estimated). (I) Base coverage of the single cell RNA-Seq assay in single ES cells. To obtain the coverage length distributions of our cDNAs, we binned all 24,435 transcripts based on their sizes, with bin n containing all transcripts of size less than n kb. Base coverage is generated for each bin of transcripts and scaled to the total number of aligned reads. The obtained distribution is represented as a function of the distance to the 3’ end of the transcripts. The read distribution for regions 3 kb away from the 3’end is very limited, which agrees with our gel results (data not show).

Table S1 The expression of 385 ESC related genes during ICM outgrowth by single cell real-time PCR.

Table S2 Single cell RNA-Seq counts (1) mapped to RefSeq for ICM, ESC and ICM outgrowth; (2) mapped to unique known exon-exon junctions and exons for each known splicing variant in RefSeq.

Table S3 Summary of RNA-Seq alignments for ICM, ESC and ICM outgrowth.

Table S4 GO analysis results of functions, networks, and pathways for (1) Refseq transcripts with FC[ESC/ICM]>4 or FC[ESC/ICM]<0.25, p<0.01); (2) Refseq transcripts with FC[Day5-Oct4$^+$/Day5-Oct4$^-$]>4 or FC[Day5-Oct4$^+$/Day5-Oct4$^-$]<0.25, p<0.01); (3) splicing variances with FC[ESC/ICM, junctions]>2 or FC[ESC/ICM, junctions]<0.5, p<0.01; (4) genes involved in Oct4 pathways, and (5) gene involved in stem cell pluripotency pathways using Ingunity systems software (www.ingenuity.com); (6) Expression changes of 114 epigenetic regulators between ICM and ESCs.

Table S5 MicroRNA expression profile of ICM from E3.5 blastocysts and ESCs.

Table S6 MicroRNA enrichment calculations using target prediction algorithms of PicTar, Miranda, or TargetScan.

Table S7 The lists of 1,378 potential pluripotency genes (FC[Day5-Oct4$^+$/Day5-Oct4$^-$]>4, p<0.01, and r >0.6). Here r represents the averaged correlation coefficient with the expression of *Oct4, Sox2,* and *Nanog* in ICM outgrowth.
